# *Cymbopogon citratus* (DC.) Stapf aqueous extract ameliorates loperamide-induced constipation in mice by promoting gastrointestinal motility and regulating the gut microbiota

**DOI:** 10.3389/fmicb.2022.1017804

**Published:** 2022-10-04

**Authors:** Xiaoyu Gao, Yifan Hu, Yafei Tao, Shuangfeng Liu, Haowen Chen, Jiayi Li, Yan Zhao, Jun Sheng, Yang Tian, Yuanhong Fan

**Affiliations:** ^1^Engineering Research Center of Development and Utilization of Food and Drug Homologous Resources, Ministry of Education, Yunnan Agricultural University, Kunming, China; ^2^College of Agronomy and Biotechnology, Yunnan Agricultural University, Kunming, China; ^3^College of Food Science and Technology, Yunnan Agricultural University, Kunming, China; ^4^Department of Science and Technology, Yunnan Agricultural University, Kunming, China; ^5^Yunnan Provincial Engineering Research Center for Edible and Medicinal Homologous Functional Food, Yunnan Agricultural University, Kunming, China; ^6^Yunnan Plateau Characteristic Agricultural Industry Research Institute, Yunnan Agricultural University, Kunming, China

**Keywords:** gut microbiota, lemon grass, constipation, gastrointestinal motility, enteric nervous system, intestinal inflammation, gut barrier, Muribaculaceae

## Abstract

Slow transit constipation (STC) is the most common type of functional constipation. Drugs with good effects and few side effects are urgently needed form the treatment of STC. *Cymbopogon citratus* (DC.) Stapf (CC) is an important medicinal and edible spice plant. The wide range of biological activities suggested that CC may have laxative effects, but thus far, it has not been reported. In this study, the loperamide-induced STC mouse model was used to evaluate the laxative effect of the aqueous extract of CC (CCAE), and the laxative mechanism was systematically explored from the perspectives of the enteric nervous system (ENS), neurotransmitter secretion, gastrointestinal motility factors, intestinal inflammation, gut barrier and gut microbiota. The results showed that CCAE not only decreased the serum vasoactive intestinal polypeptide (VIP), induced nitric oxide synthases (iNOS), and acetylcholinesterase (AchE) in STC mice but also increased the expression of gastrointestinal motility factors in colonic interstitial cells of Cajal (ICCs) and smooth muscle cells (SMCs), thereby significantly shortening the defecation time and improving the gastrointestinal transit rate. The significantly affected gastrointestinal motility factors included stem cell factor receptor (*c-Kit*), stem cell factor (*SCF*), anoctamin 1 (*Ano1*), ryanodine receptor 3 (*RyR3*), smooth muscle myosin light chain kinase (*smMLCK*) and Connexin 43 (*Cx43*). Meanwhile, CCAE could repair loperamide-induced intestinal inflammation and intestinal barrier damage by reducing the expression of the pro-inflammatory factor *IL-1*β and increasing the expression of the anti-inflammatory factor *IL-10*, chemical barrier (*Muc-2*) and mechanical barrier (*Cldn4*, *Cldn12*, *Occludin*, *ZO-1*, and *ZO-2*). Interestingly, CCAE could also partially restore loperamide-induced gut microbial dysbiosis in various aspects, such as microbial diversity, community structure and species composition. Importantly, we established a complex but clear network between gut microbiota and host parameters. Muribaculaceae, Lachnospiraceae and UCG-010 showed the most interesting associations with the laxative phenotypes; several other specific taxa showed significant associations with serum neurotransmitters, gastrointestinal motility factors, intestinal inflammation, and the gut barrier. These findings suggested that CCAE might promote intestinal motility by modulating the ENS-ICCs-SMCs network, intestinal inflammation, intestinal barrier and gut microbiota. CC may be an effective and safe therapeutic choice for STC.

## Graphical Abstract

CCAE might promote intestinal motility by modulating the ENS-ICCs-SMCs network, intestinal inflammation, intestinal barrier, and gut microbiota. 
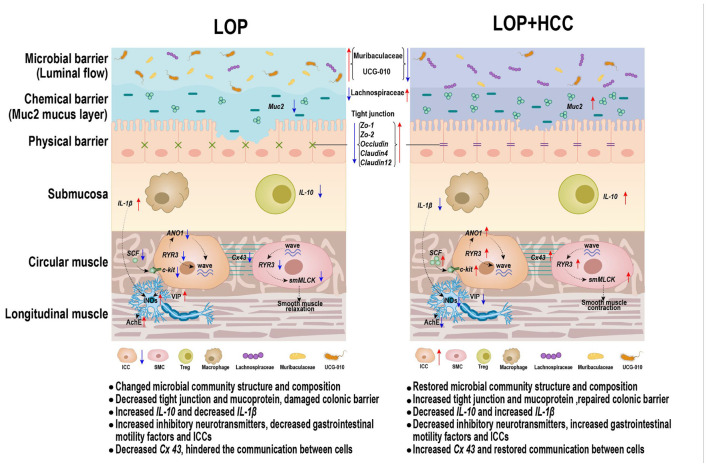

## Introduction

In recent years, due to changes in the human diet and lifestyle, constipation has become increasingly common and has gradually reduced people’s quality of life (Bharucha and Lacy, 2020). Constipation can be roughly divided into secondary constipation and functional constipation. Slow transit constipation (STC) is the most common type of functional constipation. STC is characterized by prolonged colonic transit time and reduced colonic high amplitude propulsion and contraction after eating, and the main symptoms are dry stool, difficulty in defecation, and a decrease in stool weight and frequency. At present, most scholars believe that the pathophysiological mechanism of STC may be related to nervous system diseases, abnormal hormone levels *in vivo*, smooth muscle dysfunction, abnormal interstitial cells of Cajal (ICCs), and gut microbiota imbalance.

Medications are still the main method for the treatment of STC, and they can be divided into Western medicine, traditional Chinese medicine and microbial drugs. Mainstream Western medicine therapies represented by various laxatives, prokinetic drugs and secretagogues often fail or have only short-term efficacy and induce side effects (Vriesman et al., 2020). For the treatment of STC, drugs with good effects, few side effects and clearly defined functional components are urgently needed. An innovative and efficacious drug for the treatment of STC may be found in food and natural drug resources.

*Cymbopogon citratus* (DC.) Stapf (CC) is a spice commonly used in soups and grills in Asian countries such as China, India, Thailand, Singapore, Sri Lanka and Vietnam. CC is also a traditional Chinese medicinal plant. Ancient books in China record its effects of dredging wind and collaterals, reducing swelling and pain, and gastric ventilation (Li et al., 2020). Recent scientific studies have shown that CC has antibacterial (Iram et al., 2019), antioxidant (Tiwari et al., 2010), anti-inflammatory (Figueirinha et al., 2010), anti-anxiety (Mendes Hacke et al., 2020) and antidepressant (Umukoro et al., 2017) activities, and it is beneficial in the treatment of diabetes (Borges et al., 2021), liver damage (Uchida et al., 2017), and even cancers (Rojas-Armas et al., 2020; Gomes et al., 2021; Pan et al., 2022).

Although CC has a variety of biological activities, its protective effect on the gastrointestinal tract is the most noteworthy. Volatile CC oil can effectively alleviate gastric ulcers in mice induced by absolute ethanol and aspirin (Fernandes et al., 2012; Venzon et al., 2018) and shows relatively effective inhibition of acetylcholinesterase activity (Madi et al., 2021). CC is also popular as a lemongrass tea in North and South America, West Africa and other countries and can be used to aid digestion (Kieling and Prudencio, 2019), which indicates that the water-soluble part of CC also has good gastrointestinal regulatory function. The research progress of CC in gastrointestinal regulatory activities suggests that it may be effective in relieving constipation. However, whether CC can alleviate constipation and how CC alleviates constipation is still unclear. To this end, the loperamide-induced STC mouse model was used to evaluate the laxative effect of the aqueous extract of CC, and the mechanism was systematically explored from the perspectives of the enteric nervous system (ENS), neurotransmitter secretion, gastrointestinal motility factors, gut barrier, intestinal inflammation and gut microbiota in this study.

## Materials and methods

### Preparation and chemical composition determination of *Cymbopogon citratus* (DC.) Stapf aqueous extract

Ultramicro-powder of CC leaves was obtained from a local company in Nujiang City, Yunnan Province (China). Then, 200 g ultramicro-powder was boiled for 3 min in 2 L ultrapure water (pH = 6.8). After cooling to room temperature, the extraction solution was immediately centrifuged at 5,000 rpm for 5 min. The precipitates were collected twice under the same conditions and then discarded. All the supernatants were combined and dried in vacuum freeze-drying equipment for 2–3 days. The final product was greenish brown in color, with a yield of 17.6%. The dried extract was weighed and dissolved in distilled water just before administration to experimental animals. The main nutritional components and phytochemical composition of CC aqueous extract (CCAE) were determined by various methods. The methods are described briefly in Supplementary Table 1.

### Animal experimental design

Six-week-old male KM mice (20–25 g) were purchased from Chengdu Dossy Experimental Animals Co., Ltd., China. All mice were housed in specific pathogen-free barrier conditions (24 ± 1°C, 30–50% humidity, 12-h daylight cycle, lights off at 20:00) with a normal chow diet (10.8% fat, 68.7% carbohydrates, and 20.5% protein, according to caloric intake) and water *ad libitum*. After 7 days of acclimation, the mice were divided into six groups of 12 mice according to their body weights. Three mice were housed together in each cage, and the weight, food intake, and water consumption of the mice were recorded every day. Importantly, 6 mg/kg⋅bw loperamide (LOP, mg/kg⋅body weight, Sigma) was used to generate the STC mouse model.

**FIGURE 1:**
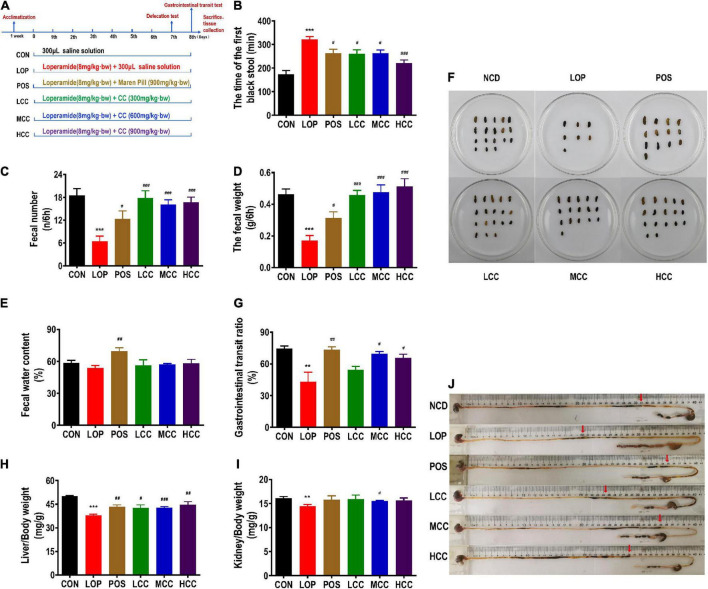
Influences of CCAE on loperamide-induced constipation symptoms in mice. **(A)** Grouping and basic workflow of animal experiment. **(B)** The defecation time of the first black stool, FBS. **(C)** Number of feces excreted in 6 h, FN. **(D)** Wet weight of feces excreted in 6 h, FW. **(E)** Fecal water content. **(F)** Representative fecal morphology of each group. **(G)** Gastrointestinal transit rate, GTR. **(H)** Liver index. **(I)** Kidney index. **(J)** Ink advancing distance and intestinal length. The data are expressed as the means ± SEMs (*n* = 10-12). *, Compared with the CON group; #, compared with the LOP group. **P < 0.01, ***P < 0.001. #*P* < 0.05, ##*P* < 0.01, ###*P* < 0.001.

The groups were designed as follows: CON group (control group, received saline solution as vehicle), LOP group (model group, received loperamide), POS group (positive control group, received 900 mg/kg⋅bw Maren pill from Beijing Tongrentang Pharmaceutical Co., Ltd, Beijing, China), LCC group (received loperamide and 300 mg/kg⋅bw CCAE, low dosage), MCC group (received loperamide and 600 mg/kg⋅bw CCAE, medium dosage) and HCC (received loperamide and 900 mg/kg⋅bw CCAE, high dosage). STC model mice were induced by administration of LOP for 8 days, and at the same time, the mice were gavaged with daily oral doses of 300 μL of solutions designated by their experimental group assignment. Required doses of the Maren pill and CCAE were dissolved in 300 μL of saline solution (Figure 1A). All mice were deprived of food but not water overnight before the defecation test and gastrointestinal transit test. All procedures were previously approved by Animal Ethics Committee of Yunnan Agriculture University.

### Defecation test

At 08:00 on the seventh day of the animal experiment, mice were given the normal dose of the drugs by gavage, the homemade ink (300 μL) was given by gavage 30 min later, and the defecation experiment of mice was officially started. The defecation time of the first black stool (FBS) of each mouse was recorded carefully, and the fecal wet weight (FW), fecal number (FN) and water content of stools were also analyzed for each mouse for 6 h from the start of the defecation test to evaluate the laxative effect.

### Gastrointestinal transit test and tissue collection

At 08:00 on the eighth day of the animal experiment, mice were administered the normal dose of the drugs. Thirty minutes later, the ink (300 μL) was given to each mouse. After 20 min, the mice were sacrificed quickly in a chamber saturated with CO_2_, the abdominal cavity was opened, and the blood was collected from the abdominal aorta. The mesentery of each gastrointestinal tract was carefully stripped, and then the length of the whole small intestine and the length marked by the ink were measured to calculate the gastrointestinal transit rate (GTR).

Meanwhile, the distal ileum, cecum, and proximal colon were accurately dissected from each mouse. The contents of the ileum and colon segments were thoroughly flushed with cold PBS to remove feces. Ceca contents were washed from the cecum in a 2-mL Eppendorf tube containing 1.0 mL cold Milli-Q water. The liver, kidney, and cecum tissues were weighed. Cleaned tissues were subsequently placed in individual cryogenic tubes. Tissues and ceca contents were all flash-frozen in liquid nitrogen and stored at −80°C until analysis.

### Enzyme-linked immunosorbent assay

After the mice were sacrificed, blood was collected immediately and incubated at 37°C for 30 min and centrifuged at 4°C at 3,500 rpm for 10 min, and serum was collected. The contents of neurotransmitters, including vasoactive intestinal polypeptide (VIP), induced nitric oxide synthases (iNOS), acetylcholinesterase (AchE), and serotonin (5-HT), in the serum of mice were determined by using an ultrasensitive enzyme-linked immunosorbent assay (ELISA) kit (Cusabio, China).

### RNA preparation and quantitative PCR analysis of gene expression

FastPure Cell/Tissue Total RNA Isolation Kits (RC101-01, Vazyme, China) were used for the total RNA extraction from mouse tissues. HiScript III RT SuperMix for qPCR (plus gDNA wiper, R323-01, Vazyme, China) was used for RNA reverse transcription, and ChamQ Universal SYBR qPCR Master Mix (Q711-02, Vazyme, China) was used for the quantitative PCR analysis of gene expression. The relative amount of the target mRNA was normalized to the *RPL-19* level, and the results were calculated by the 2^–ΔΔ*Ct*^ method. The detailed method has been described previously (Gao et al., 2017). The primer sequences are presented in Supplementary Table 2.

### Histopathological examination and immunohistochemistry assay

After the mice of each group were sacrificed, the proximal colon was collected immediately, fixed with 10% formalin, embedded in paraffin, sectioned to a thickness of 5 mm, deparaffinized and submitted to hematoxylin and eosin (H&E, Sigma-Aldrich, Shanghai, China) staining. Immunohistochemistry was performed using a previously described method (Huang et al., 2019). Briefly, 3 μm sections were deparaffinized in xylene and rehydrated in graded alcohol. After quenching endogenous peroxidase activity and blocking non-specific binding, the sections were incubated with a rabbit monoclonal antibody [EPR22566-344] against c-Kit (Abcam, ab256345) overnight at 4°C and then incubated with the secondary antibody goat anti-rabbit IgG (H + L) HRP (ab0101, Abways) at room temperature for 30 min. Finally, the slides were incubated with reagents from the Avidin-Biotin Complex Kit (Vector Laboratories, Inc., Burlingame, USA) and a 3,3′-diaminobenzidine kit (Tiangen, China) according to the manufacturer’s instructions. Images were captured with a Nikon Ci-S microscope and Nikon DS-U3 imaging system. The proportion of c-Kit-positive cells in the colonic muscle layer was determined using an image analyzer (Image-Pro Plus 6.0, Media Cybernetics, Inc., Rockville, USA).

### Ceca content DNA extraction and 16S rRNA gene sequencing

After the mice of each group were sacrificed, the contents of the cecum were collected immediately, and the total microbial genomic DNA was extracted by the E.Z.N.A.^®^ soil DNA Kit (Omega Bio-Tek, Norcross, GA, USA) according to the manufacturer’s instructions. Agarose gel electrophoresis (1.0%) and a NanoDrop^®^ ND-2000 spectrophotometer (Thermo Scientific Inc., Shanghai, United States) were used to check the quality and concentration of DNA. Qualified DNA was kept at −80°C until further use. The hypervariable region V3–V4 of the bacterial 16S rRNA gene was amplified with the primer pairs 338F (5′-ACTCCTACGGGAGGCAGCAG-3′) and 806R (5′-GGACTACHVGGGTWTCTAAT-3′) by an ABI GeneAmp^®^ 9700 PCR thermocycler (ABI, Arlington, USA). The PCR conditions and the purification of the PCR products were performed using a previously described method (Gao et al., 2020).

According to the standard protocols by Majorbio Bio-Pharm Technology Co. Ltd. (Shanghai, China), purified amplicons were pooled in equimolar amounts and paired-end sequenced on an Illumina MiSeq PE300 platform (Illumina, San Diego, CA, United States). The raw sequencing reads were deposited into the NCBI Sequence Read Archive database.

### Amplicon sequence processing and analysis

After demultiplexing, the resulting sequences were quality filtered with Fastp and merged with FLASH. Then, the high-quality sequences were de-noised using DADA2 (plugin in the QIIME2 pipeline with recommended parameters), which obtains single nucleotide resolution based on error profiles within samples. DADA2 de-noised sequences are usually called amplicon sequence variants (ASVs). To minimize the effects of sequencing depth on alpha and beta diversity measurements, the number of sequences from each sample was rarefied to 20,000, which still yielded an average Good’s coverage of 97.90%. Taxonomic assignment of ASVs was performed using the Naive Bayes consensus taxonomy classifier implemented in QIIME2 and the SILVA 16S rRNA database.

### Bioinformatics analysis

The Majorbio Cloud platform^1^ was used to analyze the gut microbiota. Based on the ASV information, rarefaction curves and alpha diversity indices, including observed ASVs, were calculated with Mothur v1.30.1. The similarity among the microbial communities in different samples was determined by principal coordinate analysis (PCoA) based on Bray-Curtis dissimilarity using the Vegan v2.5-3 package. Linear discriminant analysis (LDA) effect size (LEfSe)^2^ was performed to identify the significantly abundant taxa (phylum to genera) of bacteria among the different groups (LDA score > 2.0, *P* < 0.05).

### Statistical analysis

The data are expressed as the means ± standard errors of the means (SEMs). The unpaired two-tailed Student’s *t*-test was performed to analyze two independent groups. Bivariate correlations were calculated using Spearman’s *r* coefficients. Heatmaps were constructed using HemI 1.0 software^3^. Unless otherwise specified in the figure legends, the results were considered statistically significant at a *P*-value of < 0.05.

## Results

### Nutritional components and phytochemical composition of aqueous extract of *Cymbopogon citratus* (DC.) Stapf

The contents of the main nutritional components are shown in Supplementary Table 1. The macronutrients included protein (8.54%), carbohydrate (60.10%), fat (1.99%), water (4.06%), dietary fiber (11.79%), and crude polysaccharide (0.65%). The ash, total acid, and sodium contents were 13.52, 0.26, and 0.11%, respectively.

The phytochemical compositions of CCAE were also examined by widely targeted metabolomics (HPLC-QQQ-MS/MS). As shown in Supplementary Table 3, among the top ten chemical compound categories found in CCAE, flavonoids showed the highest relative abundance, accounting for 24.30%. Other compounds that accounted for more than 10% of the total were nucleotides (19.66%), amino acids (15.81%), organ oxygen compounds and carboxylic acids (14.85%), and alkaloids (13.51%).

There were 23 compounds with a relative abundance of more than 1% (Supplementary Table 4). Unexpectedly, the compound with the highest abundance belonged to the alkaloid betaine (13.59%). The compounds that accounted for more than 5% of the total are pyrrolidonecarboxylic acid (9.91%), vidarabine (7.45%), proline (6.50%), and homoorientin (5.74%).

### Aqueous extract of *Cymbopogon citratus* (DC.) Stapf alleviated loperamide-induced slow transit constipation symptoms

During the experiments, all mice appeared healthy and showed no abnormal behaviors. Loperamide and CCAE treatment did not show an obvious influence on body weight, food intake, or water consumption (Supplementary Figure 1). Compared with the CON group, FBS in the LOP group was significantly increased, and CCAE treatment shortened the time (Figure 1B). In addition, FW and FN in the LOP group were significantly reduced, and CCAE treatment significantly reversed these characteristics of feces (Figures 1C,D). However, CCAE did not increase the water content of the feces significantly (Figure 1E).

In the gastrointestinal transit test, the medium and high doses of CCAE (MCC and HCC groups) significantly reduced the symptoms of lower GTR induced by loperamide in mice (Figure 1G). Notably, CCAE reversed the decrease in liver and kidney indices induced by loperamide (Figures 1H,I). In general, CCAE not only alleviated a series of STC symptoms but also reversed the possible negative effects on mouse organs caused by loperamide. CCAE showed a mild and good laxative effect, which was comparable to the effect of the traditional Chinese drug Maren pill.

### Aqueous extract of *Cymbopogon citratus* (DC.) Stapf reversed the changes in serum neurotransmitters induced by loperamide

**FIGURE 2:**
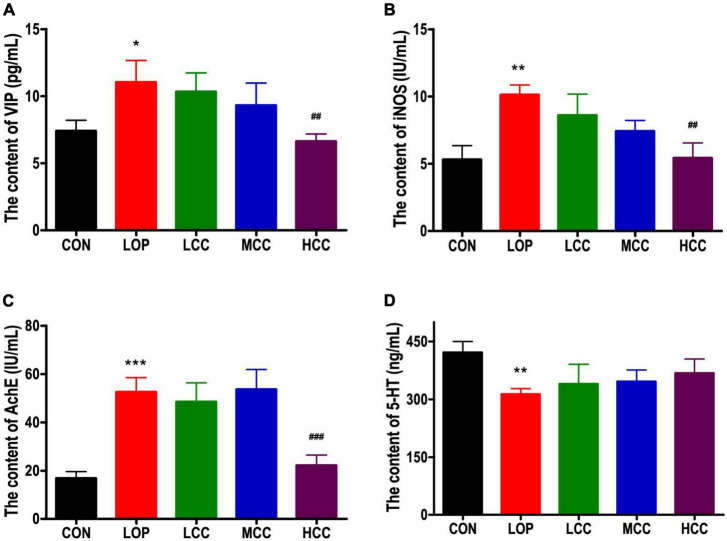
**(A)** Vasoactive intestinal polypeptide (VIP), **(B)** induced nitric oxide synthases (iNOS), **(C)** acetylcholinesterase (AchE), and **(D)** serotonin (5-HT) in serum. The data are expressed as the means ± SEMs (*n* = 8). *, compared with the CON group; #, compared with the LOP group. *P < 0.05; **P < 0.01; ***P < 0.001; ^##^*P* < 0.01; ^###^*P* < 0.001.

We investigated whether loperamide-induced defecation delay were accompanied by alterations in the molecular regulators for STC, including iNOS, AchE, VIP, and 5-HT, because they are associated with the proliferation of interstitial cells of Cajal and gastrointestinal mobility. Similar expression patterns were observed for VIP, iNOS, and AchE. These levels were significantly increased only in the LOP, whereas the HCC mice showed reduced levels of these molecules (Figures 2A–C). However, the neurotransmitter 5-HT did not have a significant reversal trend in the CCAE treatment groups. In general, LOP increased the serum level of inhibitory neurotransmitters and AchE, and the high dosage of CCAE (HCC group) reduced them to the control level.

### Aqueous extract of *Cymbopogon citratus* (DC.) Stapf enhanced the reduced gastrointestinal motility factors induced by loperamide

**FIGURE 3:**
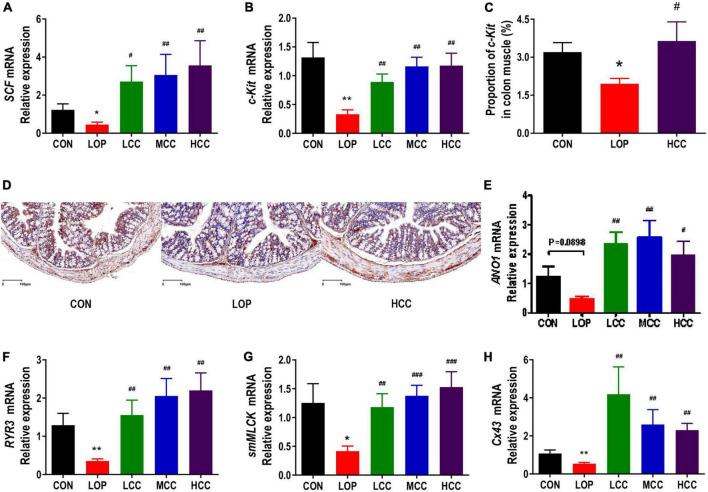
Aqueous extract of *Cymbopogon citratus* (DC.) Stapf (CCAE) increased the expression of gastrointestinal motility factors in the colon of STC mice. **(A,B)** The mRNA expression of stem cell factor (*SCF*) and stem cell factor receptor (*c-Kit*) in the colon. **(C)** Percentage of c-Kit-positive area in colon muscle. **(D)** Representative immunostaining images of colon sections stained for c-Kit. **(E–H)** The mRNA expression of *Anoctamin 1* (*Ano1*), *Ryanodine receptor 3* (*RyR3*), *smooth muscle myosin light chain kinase* (*smMLCK*), and *Connexin 43* (*Cx43*) in the colon. The data are expressed as the means ± SEMs (*n* = 8). *, Compared with the CON group; #, compared with the LOP group. *P < 0.05, **P < 0.01, ***P < 0.001. #*P* < 0.05, ##*P* < 0.01, ###*P* < 0.001.

Interstitial cells of Cajal (ICCs) and smooth muscle cells (SMCs) play a very important role in regulating gastrointestinal motility. The contraction and relaxation of smooth muscle is controlled by the slow wave of smooth muscle. As the pacemaker cells of smooth muscle and mediators of neurotransmitters, ICCs exist in the muscle layer. Stem cell factor receptor (c-Kit) is a biomarker of ICC. The stem cell factor (SCF) and c-Kit signaling pathways play vital roles in maintaining the development, differentiation and phenotype of ICCs. In mice in the LOP group, loperamide significantly reduced the mRNA expression of *c-Kit* and *SCF* in the colon; interestingly, CCAE treatment significantly increased their expression (Figures 3A,B). The immunohistochemistry results showed that c-Kit expression in colon muscle was decreased with the onset of STC induced by loperamide and increased with CCAE treatment (Figures 3C,D).

In ICCs, calcium channels and calcium-activated chloride channels are mainly used to generate Ca^2+^ transients and then generate slow-wave currents, which are transmitted to smooth muscle cells through the network structure. The opening up of ryanodine receptor 3 (RyR3) in calcium channels can increase the release of Ca^2+^, activate myosin light chain kinase (MLCK), and finally cause smooth muscle contraction. Anoctamin 1 (Ano1), a calcium-activated chloride channel, also exists in ICCs. Connexin 43 (Cx43) is the most important connexin constituting gap junctions, which widely exist between ICCs and SMCs and play an important role in slow wave transmission. Mutation and reduction of Cx43 affect the number of gap junction channels on the cell membrane, thereby hindering the transmission of intercellular signals and resulting in gastrointestinal motility dysfunction. In this study, we found that loperamide significantly or nearly significantly reduced the mRNA expression levels of *Ano1*, *RyR3*, *Cx43* and smooth muscle myosin light chain kinase (*smMLCK*), while CCAE significantly restored the expression levels of these genes to varying degrees, even exceeding the expression levels of the CON group (Figures 3E–H). In general, CCAE might promote defecation in STC mice by increasing the expression of key gastrointestinal motility factors present in colonic ICCs and SMCs.

### Aqueous extract of *Cymbopogon citratus* (DC.) Stapf improved the intestinal inflammation and intestinal barrier damage induced by loperamide

**FIGURE 4:**
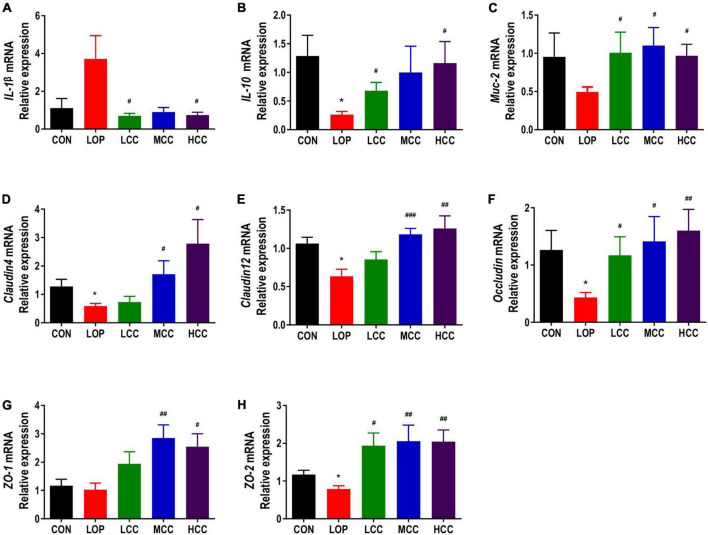
Effects of CCAE administration on the mRNA expression of inflammation- and intestinal barrier-related factors in the colons of mice. **(A–H)** *IL-1*β, *IL-10*, *Muc-2*, *Cldn4*, *Cldn12*, *Occludin*, *ZO-1*, and *ZO-2*. The data are expressed as the means ± SEMs (*n* = 8). *, Compared with the NCD group; #, compared with the LOP group. *P < 0.05, **P < 0.01, ***P < 0.001. #*P* < 0.05, ##*P* < 0.01, ###*P* < 0.001.

Intestinal barrier function is essential for maintaining intestinal homeostasis. Dysfunction of the intestinal barrier may trigger an excessive immune response and prolong the inflammatory state, resulting in a variety of gastrointestinal diseases. The mRNA expression of *IL-1*β in the LOP group was higher than that in the CON group, while CCAE treatment significantly reduced it (Figure 4A). However, loperamide and CCAE treatment did not have an obvious influence on the mRNA expression of *MCP-1*, *TNF*-α or *IL-6* (Supplementary Figures 2A–C). Interestingly, the gene expression of the anti-inflammatory factor *IL-10* was significantly inhibited by loperamide, and CCAE effectively restored the expression level of *IL-10* in the colon of the STC model mice (Figure 4B). The regulatory effect on iNOS also suggests that CCAE has a certain anti-inflammatory ability (Figure 2B).

The mucus layer is mainly lined with the muco-protein (Muc) skeleton and complex O-linked oligosaccharides, which can separate the bacteria in the intestinal lumen from the intestinal epithelial cells and allow the absorption of nutrients. We found that the mRNA expression of *Muc-2* in the LOP group was inhibited, and CCAE treatment could prevent this inhibition and restore the expression of *Muc-2* to the control level (Figure 4C).

Claudin, Occludin, and ZO family proteins play an important role in maintaining the normal physiological functions of epithelial cells. The mRNA expression of tight junction proteins, including *Claudin4*, *Claudin12*, *Occludin*, *ZO-1*, and *ZO-2*, showed similar trends among the groups, all of which were significantly lower in the LOP group than in the CON group, and the CCAE treatment groups had higher levels than the LOP group (Figures 4D–H). In general, CCAE showed a good ability to prevent loperamide-induced intestinal inflammation and impaired barrier function.

### Aqueous extract of *Cymbopogon citratus* (DC.) Stapf partially restored loperamide-induced gut microbial dysbiosis

**FIGURE 5:**
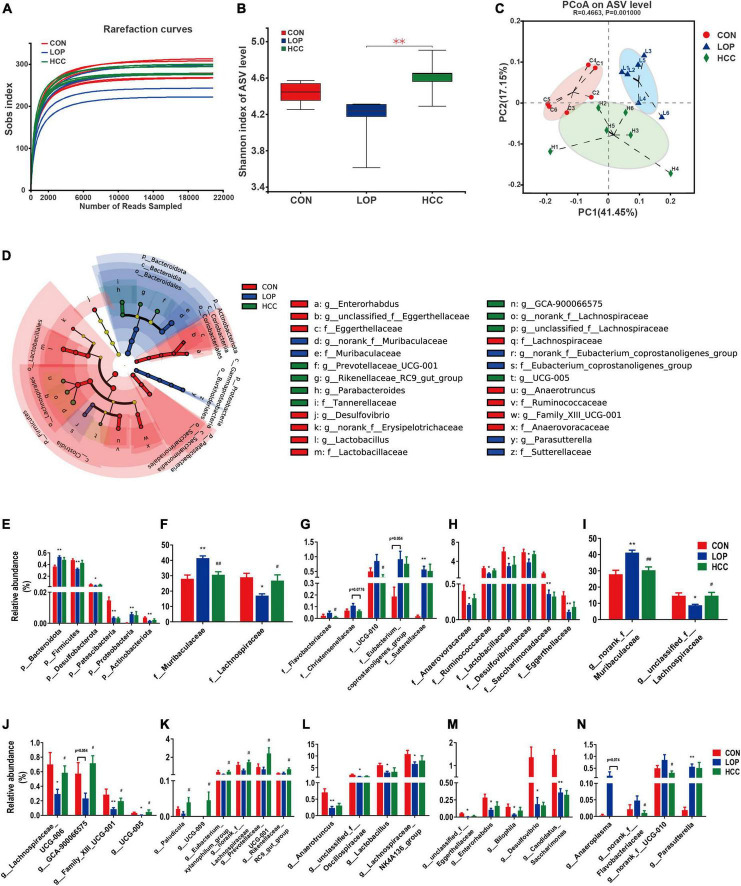
HCC restores the LOP-induced gut microbial community structural and compositional shift. **(A)** The rarefaction curve of the Sobs index of each sample plateau at the ASV level. **(B)** Alpha diversity estimated by the Shannon index. **(C)** PCoA (principal coordinate analysis) plot based on weighted UniFrac distance. **(D)** Linear discriminant analysis effect size (LEfSe) analyses (LDA score of > 2.0). **(E–N)** Relative abundances of gut microbiota at the phylum, family, and genus levels, which were significantly affected by LOP or HCC, especially those reversed by HCC treatment. The data are expressed as the means ± SEMs (*n* = 6). *, Compared with the CON group; #, compared with the LOP group. *P < 0.05, **P < 0.01. #*P* < 0.05, ##*P* < 0.01.

The gut microbiota plays an important role in the progression of STC. The V3–V4 regions of the 16S rRNA genes were sequenced to determine the effect of CCAE (HCC group) on the STC model mice. We obtained 532,597 sequences in a total of 18 samples from the CON, LOP, and HCC groups, each with more than 21,571 valid sequences for subsequent taxonomic analysis. Through systematic bioinformatics analysis, we identified 591 ASVs, 84 genera, 40 families, and 9 phyla. The rarefaction curve of the Sobs index of each sample plateau with the current sequencing indicated that the sequencing result was credible (Figure 5A).

The community diversity (Shannon, Sobs, Chao, and Ace) and community evenness (Shannoneven, Simpsoneven) indices were all reduced in the LOP group compared with the CON group (Figure 5B and Supplementary Figure 3). CCAE administration reversed these loperamide-induced diversity index changes to varying degrees. Notably, CCAE administration significantly improved the loperamide-induced decrease in the Shannon, Shannoneven, and Simpsoneven indices (*P* < 0.05, Figure 5B and Supplementary Figures 3A,B). These results indicated that CCAE could increase the α diversity of the gut microbiota in STC mice. CCAE also altered β diversity in STC mice (Figure 5C). Although the administration of high-dose CCAE could not completely reverse the significant changes induced by loperamide, CCAE still appeared to regulate the abnormal gut microbiota in STC mice. Moreover, CCAE changed the cecal microbial composition of STC mice (Supplementary Figures 4A–C), and the microbial composition of CCAE-treated mice was clustered with that of the CON group (Supplementary Figures 4D–F).

Linear discriminant analysis (LDA) effect size (LEfSe) analyses were used to obtain the dominant microbiota at different levels for each group (Figure 5D and Supplementary Figures 5A–D). Here, a total of 43 different taxa from the 3 groups are displayed, including 5 phyla, 5 classes, 6 orders, 10 families, and 17 genera (Supplementary Figures 5A–C). We focused on the taxa that were significantly affected by LOP or HCC, especially those significantly changed by HCC treatment (Supplementary Figure 5D).

At the phylum level (Figure 5E), the relative abundances of six phyla were all significantly altered by loperamide treatment, including Firmicutes, Bacteroidota, Proteobacteria, Actinobacteriota, Desulfobacterota, and Patescibacteria. This finding suggests a comprehensive effect of loperamide on the gut microbiota in mice. CCAE treatment had different degrees of reversal effects on five phyla, except for Patescibacteria, but these reversal effects did not reach statistical significance (Figure 5E).

At the family level, the dominant families of gut bacteria, Muribaculaceae and Lachnospiraceae, belonging to Firmicutes and Bacteroidota, respectively, were significantly upregulated and downregulated in relative abundance under loperamide induction, respectively; importantly, CCAE could significantly reverse the loperamide-induced changes (Figure 5F). Similarly, CCAE significantly reduced the relative abundance of Flavobacteriaceae and UCG-010 compared to the LOP group, although loperamide was not able to increase their abundances significantly (Figure 5G). There were also some other families that were significantly upregulated or downregulated under the induction by loperamide, and CCAE treatment had a certain reversal effect on them, but there was no statistical significance (Figures 5G,H). Among them, Eubacterium coprostanoligenes group and Sutterellaceae were significantly upregulated (Figure 5G); Desulfovibrionaceae, Lactobacillaceae, Ruminococcaceae, Saccharimonadaceae, Anaerovoracaceae, and Eggerthellaceae were significantly downregulated (Figure 5H).

At the genus level, the most interesting taxa still belonged to Firmicutes and Bacteroidota. *Unclassified_f__Lachnospiraceae*, *Lachnospiraceae_UCG-006* and *GCA-900066575* belong to Lachnospiraceae, and their relative abundances were all significantly reduced in mice of the LOP group but reversed significantly in the HCC group (Figures 5I,J). Notably, *norank_f__Muribaculaceae* (belonging to Bacteroidota) and *Anaeroplasma* (belonging to Firmicutes) showed the opposite variation (Figures 5I,N). Some other genera in Firmicutes also showed nearly the same patterns as Lachnospiraceae, including Family_XIII_UCG-001 and *UCG-005* (Figure 5J). CCAE treatment significantly increased the relative abundance of *Paludicola*, *UCG-009*, *Eubacterium xylanophilum group*, and *norank_f__Lachnospiraceae*, although loperamide did not reduce their abundances significantly. Interestingly, *Prevotellaceae_UCG-001* and *Rikenellaceae_RC9_gut_group* (belonging to Bacteroidota) showed a similar pattern with Lachnospiraceae (Figure 5K). *Anaerotruncus*, *unclassified_f__Oscillospiraceae*, *Lactobacillus*, and *Lachnospiraceae_NK4A136_group* were also significantly reduced by loperamide, while the reversal effects of CCAE did not reach statistical significance (Figure 5L). The same pattern was exhibited in some genera belonging to Actinobacteriota, Desulfobacterota and Patescibacteria, including *unclassified_f__Eggerthellaceae*, *Enterorhabdus*, *Bilophila*, *Desulfovibrio*, and *Candidatus_Saccharimonas* (Figure 5M). In addition, loperamide treatment enhanced the relative abundance of *norank_f__Flavobacteriaceae*, *norank_f__UCG-010* and *Parasutterella* to varying extents; CCAE treatment significantly reduced the abundance of the first two of them (Figure 5N). These results fully demonstrated that CCAE could partially restore loperamide-induced gut microbial dysbiosis in many aspects, such as microbial diversity, community structure, and species composition.

### Correlations between specific gut bacteria and core host parameters

To further clarify the possible role of gut microbiota in the amelioration of loperamide-induced STC progression by CCAE, we systematically analyzed the correlations between taxa-specific gut bacteria and core host parameters, such as laxative phenotypic indicators, serum neurotransmitters, gastrointestinal motility factors, intestinal inflammation, and intestinal barrier function, at the phylum, family, and genus levels.

**FIGURE 6:**
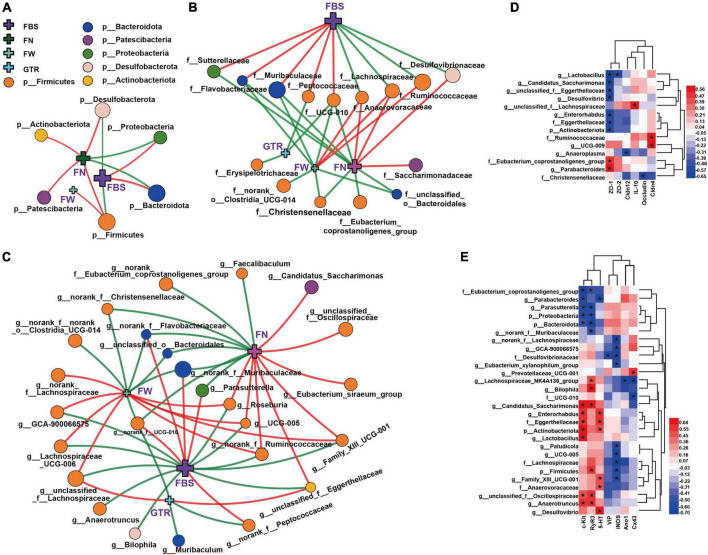
Network and heatmaps showing correlations between specific gut bacteria and core host parameters in STC mice. **(A–C)** Two-factor correlation network analysis (*P* < 0.05; Spearman, *n* = 6 in each group). Red lines represent *r* values ≥ 0.4, and green lines represent *r* values ≤ 0.4. Correlations between gut bacteria and the laxative phenotypic indicators, including **(A)** at the phylum level, **(B)** at the family level, and **(C)** at the genus level. FBS, the defecation time of the first black stool; the wet weight of the feces (FW) and the number of feces excreted in 6 h (FN); the gastrointestinal transit rate (GTR). **(D,E)** Bivariate correlations (*P* < 0.05, *n* = 6 in each group), including correlations between gut bacteria and intestinal inflammation, gut barrier function in the colon of mice **(D)**, correlations between gut bacteria and serum neurotransmitters, gastrointestinal motility factors **(E)**. *IL-10*, *ZO-1*, *ZO-2*, *Cldn4*, *Cldn12*, *Occludin*, *c-Kit*, *SCF*, *Ano1*, and *Cx43* indicate their mRNA expression levels in the colons of mice. 5-HT, iNOS, and VIP indicate their expression levels in serum. The color at each intersection indicates the value of the *r* coefficient; *P* -alues were adjusted for multiple testing according to the *Bonferroni and Hochberg* procedures. * Indicates a significant correlation between these two parameters.

Correlations between the specific gut bacteria and the laxative phenotypic indicators are shown in Figures 6A–C and Supplementary Figure 6. FBS is the core host parameter that could directly reflect the laxative effect of CCAEs on mice. At the phylum level (Figure 6A), correlations between Firmicutes, Bacteroidota and FBS showed opposite trends. Firmicutes and Desulfobacterota were significantly negatively correlated with FBS, and Bacteroidota and Proteobacteria were significantly positively correlated with FBS. Actinobacteriota and Patescibacteria were significantly positively correlated with FN.

At the family level, Anaerovoracaceae and Lachnospiraceae were significantly negatively correlated with FBS and positively correlated with FN and FW. Muribaculaceae, Flavobacteriaceae, and UCG-010 show completely opposite correlations. Ruminococcaceae and Desulfovibrionaceae were also significantly negatively correlated with FBS, while Sutterellaceae and Peptococcaceae also showed a positive correlation with FBS. Notably, there was also a significant negative correlation between UCG-010 and GTR.

At the genus level, correlations between the microbial groups and defecation phenotype are relatively complex, but the correlation laws are still clear. The genera with a strong correlation mainly belonged to Firmicutes and Bacteroidota. The important genera with a significant negative correlation with FBS mainly included *unclassified_f__Lachnospiraceae*, *Lachnospiraceae_UCG-006*, *norank_f__Ruminococcaceae*, *UCG-005*, *Roseburia*, *unclassified_f__Eggerthellaceae*, and *GCA-900066575*; meanwhile, they showed a significant positive correlation with FN or FW. The important genera with a significant positive correlation with FBS mainly included *norank_f__Muribaculaceae*, *norank_f__Flavobacteriaceae*, *Parasutterella*, and *norank_f__UCG-010*; meanwhile, they showed a significant negative correlation with FN or FW. It is worth mentioning that *unclassified_f__Eggerthellaceae* (belonging to Actinobacteriota) also exhibited a strong negative correlation with FBS and a positive correlation with GTR and FN. Interestingly, GTR showed significant correlations with a few genera, only negatively correlated with *norank_f__UCG-010*, *Muribaculum*, and *norank_f__Peptococcaceae* and positively correlated with *unclassified_f__Eggerthellaceae* and *unclassified_f__Lachnospiraceae.* Of course, some other genera of gut bacteria also showed strong associations with mouse laxative phenotypes, such as FN and FW. All these numerous correlations suggest that gut microbiota might play an important role in regulating the laxative phenotype.

Correlations between the specific gut bacteria and intestinal inflammation and gut barrier function are shown in Figure 6D. Interestingly and importantly, the anti-inflammatory factor *IL-10* was only significantly positively correlated with the highly abundant genus *unclassified_f__Lachnospiraceae*; moreover, both *unclassified_f__Lachnospiraceae* and *IL-10* were significantly reversed by CCAE in STC mice. Among the gut barrier function factors, *ZO-1* showed the most correlations with the microbial taxa. Several taxa of Actinobacteria had a strong negative correlation with *ZO-1*, and the Eubacterium_coprostanoligenes_group of Firmicutes had a strong positive correlation with *ZO-1*. Ruminococcaceae and *UCG-009* also showed a strong positive correlation with *Cldn4*. This suggests that some specific groups of Firmicutes and Actinobacteria might play important and positive roles in CCAE against loperamide-induced colonic inflammation and impaired barrier function.

The heatmap in Figure 6E clearly shows the correlations between the specific gut bacteria and serum neurotransmitters and gastrointestinal motility factors. *c-kit, RyR3*, and *iNOS* showed more correlations with gut microbes. At the family and genus levels, *Eubacterium_coprostanoligenes_group*, *Parabacteroides*, *Parasutterella*, and *norank_f__Muribaculaceae* were negatively correlated with *c-kit* and *RyR3*, while *Candidatus_Saccharimonas*, *Enterorhabdus*, Eggerthellaceae, *Lactobacillus*, *unclassified_f__Oscillospiraceae*, and *Anaerotruncus* exhibited the opposite correlation. Except for Desulfovibrionaceae, the taxa with significant negative correlations with iNOS all belong to Firmicutes, mainly including *norank_f__Lachnospiraceae*, *GCA-900066575*, Lachnospiraceae, *Eubacterium_xylanophilum_group*, *Paludicola*, *UCG-005*, and *Family_XIII_UCG-001*, in which *norank_f__Lachnospiraceae* and GCA-900066575 belong to Lachnospiraceae. Notably, the genus *Lachnospiraceae_NK4A136_group* and family UCG-010 were negatively correlated with gap junction *Cx43*, which also belongs to Firmicutes. Interestingly, the genus *Prevotellaceae_UCG-001* showed a significantly positive correlation. These results implied that some specific groups of Firmicutes, Bacteroidota, and Actinobacteriota might play different and important roles in CCAE regulating gastrointestinal peristalsis in STC mice.

## Discussion

Prolonged constipation often produces a variety of adverse reactions, causing serious distress to the affected people. Loperamide is an opioid receptor agonist that is used for the treatment of acute and chronic diarrhea caused by various etiologies. Therefore, it is widely used to induce constipation in animals (Hu et al., 2021). Loperamide mainly inhibits intestinal motility by blocking calcium channels, inhibiting calmodulin, reducing cell bypass permeability, and reducing the release of acetylcholine from intestinal nerve endings (Baker, 2007). In this study, loperamide also showed an excellent ability to shape the STC model.

Intestinal homeostasis plays an extremely important role in intestine-related diseases. Studies have shown that loperamide-induced STC animals generally have impaired intestinal homeostasis (Lin et al., 2021). Therefore, we systematically evaluated the effect of CCAE on loperamide-induced STC from three aspects, including intestinal movement, intestinal barrier and gut microbiota. We found that CCAE not only significantly decreased the expression of VIP, iNOS, and AchE (Figure 2), but also significantly increased the expression of the gastrointestinal motility factors *SCF*, *c-Kit*, *Ano1*, *RyR3*, and *smMLCK* (Figure 3), thereby improving the gastrointestinal transport rate and shortening the defecation time (Figure 1). At the same time, CCAE decreased the mRNA expression of the inflammatory factor *IL-1*β and increased the mRNA expression of the anti-inflammatory factor *IL-10*, chemical barrier *Muc-2*, mechanical barrier *Cldn4*, *Cldn12*, *Occludin*, *ZO-1*, and *ZO-2* to repair the gut barrier and maintain intestinal homeostasis (Figure 4). Interestingly, CCAE also changed the intestinal microbial community structure and composition in loperamide-induced STC mice, and some important taxa of gut microbiota were significantly regulated by CCAE (Figure 5).

In recent years, the above neurotransmitters and gastrointestinal functional factors have received increasing attention in the research of STC, but there are few comprehensive reports that connect the important factors of each part of the ENS-ICCs-SMCs network. The ENS-ICCs-SMCs network is the basic functional unit of gastrointestinal movement, and it is mainly organized through the functions of ICCs. As a pacemaker cell for gastrointestinal activity, ICCs are also promoters of gastrointestinal electrical activity transmission and regulators of neurotransmitter transmission (Zhu et al., 2021). There are a large number of neurotransmitter receptors on the cell membrane of ICCs. When some neurotransmitters bind to the corresponding nerve receptors on ICCs, they can transmit excitatory or inhibitory nerve signals to SMCs through ICCs to regulate the relaxation and contraction of smooth muscle (Kim et al., 2006, 2013; Choi et al., 2010; Cipriani et al., 2011; Liu et al., 2020). Our results indicated that CCAE could reverse the loperamide-induced decrease in the expression of ICC cell marker c-Kit and changes in serum neurotransmitters to a certain extent. The pacing effect of ICCs on SMCs depends on the generation of slow waves, which are generated by Ca^2+^-induced potential changes in ICCs (Drumm et al., 2019). In this study, the mRNA expression of *RyR3* of calcium channels and *Ano1* of calcium-activated chloride channels was significantly inhibited in the LOP group, which was significantly reversed by CCAE. After the slow wave is generated, it is transmitted to SMCs via Cx43 to contract smooth muscle (Sancho et al., 2011). smMLCK is a key regulatory enzyme that controls the initiation of smooth muscle contraction and is widely present in smooth muscle (Zhang et al., 2016). We found that loperamide could significantly inhibit the expression of *smMLCK* and *Cx43* in the mouse colon, while CCAE could significantly upregulate them. Therefore, we believe that CCAE can alleviate loperamide-induced STC by regulating the ENS-ICCs-SMCs network.

Intestinal homeostasis is mainly maintained by intestinal barrier function (Salinas et al., 2021). The normal intestinal mucosal barrier is mainly composed of four parts: mechanical barrier, chemical barrier, immune barrier and biological barrier. The occludin family, claudin family and ZO family are important protein molecules that constitute the tight junctions between cells (Zhang J. et al., 2021). The basic structure of the chemical barrier is the mucus layer, which is mainly composed of mucus and other substances secreted by the intestinal epithelium (Lu et al., 2021). When the mechanical barrier and chemical barrier are dysfunctional, bacteria and toxic products enter the immune barrier and induce production of inflammatory factors (Stolfi et al., 2022). The mRNA expression levels of *ZO-1*, *Occludin*, *Cldn1*, and *Muc-2* could be significantly downregulated in the colon of constipated animals, and their expression levels were significantly upregulated after probiotic treatment (Eor et al., 2019; Lee et al., 2019; Lu et al., 2021). CCAE can also repair the intestinal barrier and maintain intestinal homeostasis by enhancing the mRNA expression levels of chemical barriers (*Muc-2*), mechanical barriers (*Cldn4*, *Cldn12*, *Occludin*, *ZO-1*, and *ZO-2*) and anti-inflammatory factors (*IL-10*).

The gut microbiota is an important part of the intestinal barrier and belongs to the biological barrier. The importance of the gut microbiota to health is now well known. Accumulating evidence supports the critical role of gut microbiota in regulating gut motility (Ohkusa et al., 2019; Zhang S. et al., 2021). Studies have confirmed that bacterial colonization in the gut is critical for the development and maturation of the ENS (Joly et al., 2021). Abnormal gastrointestinal microbiota composition may lead to disruption of “microbiota-gut-brain axis” signaling, leading to altered gut motility (Kennedy et al., 2012; Carabotti et al., 2015). Metabolites of gut microbiota could stimulate the ENS and affect gut motility (Barbara et al., 2005). Therefore, altering the gut microbiota may affect defecation behavior by regulating intestinal motility and secretion. In this study, CCAE partially reversed the gut microbiota changes induced by loperamide.

According to the analysis results of gut microbiota, we speculate that some high-abundance families and genera might play vital roles in the process of CCAE alleviating STC. For example, Muribaculaceae, Lachnospiraceae, *norank_f__Muribaculaceae*, and *unclassified_f__Lachnospiraceae* (Figures 5F,I) not only showed significant reversal-type changes during CCAE treatment but were also significantly associated with the laxative phenotypes, serum neurotransmitters, gastrointestinal motility factors, intestinal barrier, and intestinal inflammation (Figure 6). A series of previous clinical research results confirmed the rationality of our findings and speculations in mice. For example, a clinical study of irritable bowel syndrome showed that a higher Firmicutes to Bacteroidetes ratio was positively associated with loose stools in patients (Hollister et al., 2020). Parthasarathy et al. (2016) found that genera from Bacteroidetes were more abundant in the colonic mucosal microbiota of patients with constipation, and that the profile of the fecal microbiota was associated with colonic transit; genera from Firmicutes correlated with faster colonic transit; there was a decrease in the proportion of Firmicutes and an increase in Bacteroidetes in subjects with functional constipation, while ID-HWS1000 (composed of probiotics and prebiotics) directly improved the discomfort associated with bowel movements, decreased the proportion of Lachnospiraceae, and increased the proportion of Bacteroidaceae (Kim M. C. et al., 2021).

Previous animal experimental studies also agreed with our findings. Phlorotannins derived from *Ecklonia cava* could improve the constipation phenotype and restore the abundance of Muribaculaceae in the fecal microbiota of STC rats (Kim J. E. et al., 2021). Goji Berry and soluble fiber dextrin from tapioca promote the growth of butyrate-producing bacteria, including Lachnospiraceae and Ruminococcaceae, while reducing proinflammatory factors in IL-10-deficient mice (Valcheva et al., 2015; Kang et al., 2018). We found that both *unclassified_f__Lachnospiraceae* and *IL-10* were significantly reversed by CCAE in STC mice, and *IL-10* was only significantly positively correlated with the highly abundant genus *unclassified_f__Lachnospiraceae*.

Although the abovementioned high abundance families and genera performed well in the correlation analysis with the phenotypic indicators of constipation, they generally performed well in the correlation analysis with serum neurotransmitters, gastrointestinal motility factors and intestinal barrier. In contrast, the comprehensive performance of some low abundance taxa is remarkable, including Flavobacteriaceae, UCG-010, Anaerovoracaceae, *norank_f__Flavobacteriaceae*, *Lachnospiraceae_UCG-006*, *GCA-900066575*, *Family_XIII_UCG-001*, *UCG-005*, *Paludicola*, *UCG-009*, and *Prevotellaceae_UCG-001*, although some of these taxa did not show the most prominent abundance changes in this study. We could hardly find literature reports of these taxa related to constipation. Only Lachnospiraceae_UCG-006 was found to be associated with yellow tea extract interventions for constipation relief (Cao et al., 2021), which is consistent with our findings. In addition, several low abundance taxa of Actinobacteria (*unclassified_f__Eggerthellaceae*, Eggerthellaceae and *Enterorhabdus*) had a strong correlation with intestinal barrier and gastrointestinal motility factors in this study. The correlation between the taxa and intestinal barrier has been mentioned in previous studies (Chen et al., 2021; Han et al., 2021), but there is no report on their association with gastrointestinal motility factors. This also suggests that our study may reveal more potential associations between microbes and constipation-related markers that have not yet been addressed.

Overall, we believe that gut microbiota play a very important role in regulating the laxative phenotype; some specific taxa of Firmicutes and Actinobacteria might play positive roles in CCAE against loperamide-induced colonic inflammation and impaired barrier function; some specific taxa of Firmicutes, Bacteroidota and Actinobacteriota might play different and important roles in CCAE regulating gastrointestinal peristalsis.

CCAE is a nutrient-rich, phytochemically diverse complex (Supplementary Tables 1, 3,4). Although we have systematically evaluated the laxative effect of CCAE, the laxative active components and *in vivo* pharmacodynamic substance of CCAE are still unclear. However, some main components of CCAE exhibit laxative-related biological activity. For example, dietary fiber intake can obviously increase stool frequency in patients with constipation (Yang et al., 2012); as a metabolite, the betaine content decreased in constipated rats and increased significantly after the symptoms of constipation were relieved (Kim et al., 2019); betaine can also ameliorate intestinal injury in heat-challenged broilers by suppressing inflammatory responses and enhancing mucosal barrier function (Alhotan et al., 2021); vitexin exerted neural protective effects via antioxidant, anti-inflammatory and gut microbiota modulating properties (Li et al., 2021); and crotonoside, cordycepin, and cynaroside have significant anti-inflammatory effects (Lin et al., 2020; Pei et al., 2021; Tan et al., 2021). These results on major bioactive components of CCAE not only provide evidence for our research results of CCAE relieving constipation but also provide references for us to further clarify the molecular basis how CCAE relieves constipation.

## Conclusion

In summary, we systematically studied the effect of CCAE on host parameters and the gut microbiota in loperamide-induced STC mice. We found that CCAE could significantly improve loperamide-induced constipation symptoms. We believe that CCAE might promote intestinal motility by modulating the ENS-ICCs-SMCs network, intestinal inflammation, intestinal barrier and gut microbiota, thereby relieving constipation. Meanwhile, the established correlation networks between the gut microbiota and the laxative phenotypic indicators in STC mice provided a foundation for further clarifying the relationship between the gut microbiota and host metabolism in STC mice.

## Data availability statement

The data presented in this study are deposited in the SRA repository, accession number PRJNA840843.

## Ethics statement

The animal study was reviewed and approved by Animal Ethics Committee of Yunnan Agriculture University.

## Author contributions

XG: conceptualization, data curation, formal analysis, methodology, and writing – original draft, review and editing. YH: data curation, formal analysis, methodology, visualization, and writing – original draft. YTa, SL, HC, and JL: methodology. YZ: visualization. JS: conceptualization and funding acquisition. YTi: conceptualization and supervision. YF: resources, supervision, and funding acquisition. All authors read and approved the final manuscript.

## Footnotes

1
https://cloud.majorbio.com

2
http://huttenhower.sph.harvard.edu/LEfSe

3
http://hemi.biocuckoo.org/down.php

## Supplementary material

Supplementary Figure 1 Effects of CCAE on loperamide-induced constipation symptoms in mice. **(A)** The body weight. **(B)** Food intake. **(C)** The water consumption. **(D)** Spleen index. **(E)** The small intestine length. **(F)** Cecum index.

Supplementary Figure 2 Gene expression of intestinal inflammatory factors in colon of STC mice. **(A–C)** Inflammatory factor *MCP-1*, *TNF*-α, and *IL-6*.

Supplementary Figure 3 **(A–F)** Shannoneven, simpsoneven, ace, chao, sobs, simpson index of ASV level. *compared with the NCD group, using the Kruskal-Wallis *H* test. ***P* < 0.01.

Supplementary Figure 4 Effect of CCAE on the cecum microbial composition in STC mice. **(A)** Phylum level. **(B)** Family level. **(C)** Genus level. **(D–F)** Cluster heatmaps of gut microbiota in different groups.

Supplementary Figure 5 Linear discriminant analysis effect size (LEfSe) analyses (LDA score > 2.0). **(A–C)** LEfSe analyses based on the CON, LOP and HCC groups. **(A)** At the phylum, class and order level. **(B)** At the family level. **(C)** At the genus level. **(D)** LEfSe analyses based on the LOP and HCC groups, from the phylum level to the genus level.

Supplementary Figure 6 Heat maps showing correlations between specific gut bacteria and the core laxative phenotypic indicators in STC mice. Bivariate correlations (*P* < 0.05, *n* = 6 in each group), including correlations between gut bacteria and the core laxative phenotypic indicators. FBS, the defecation time of the first black stool; the fecal wet weight (FW) and the fecal number (FN) in 6 h; the gastrointestinal transit rate (GTR). The color at each intersection indicates the value of the *r* coefficient; *P*-values were adjusted for multiple testing according to the *Bonferroni and Hochberg* procedures. * indicates a significant correlation between these two parameters (*P* < 0.05).

## Abbreviations

STC: slow transit constipation
CC: *Cymbopogon citratus* (DC.) Stapf
CCAE: aqueous extract of *Cymbopogon citratus* (DC.) Stapf
ENS: enteric nervous system
VIP: vasoactive intestinal polypeptide
iNOS: induced nitric oxide synthases
AchE: acetylcholinesterase
5-HT: serotonin
ICCs: interstitial cells of Cajal
SMCs: smooth muscle cells
c-Kit: stem cell factor receptor
SCF: stem cell factor
Ano1: anoctamin 1
RyR3: ryanodine receptor 3
smMLCK: smooth muscle myosin light chain kinase
Cx43: connexin 43
FBS: the defecation time of the first black stool
FW: fecal wet weight
FN: fecal number
GTR: gastrointestinal transit rate
TNF- α: tumor necrosis factor-alpha
IL-1 β: interleukin-1 β
IL-6: interleukin-6
IL-10: interleukin-10
MCP-1: monocyte chemotactic protein-1
Muc2: mucin 2
Occludin: occluding
ZO-1: zonula occludens protein 1
ZO-2: zonula occludens protein 2
Cldn4: claudin 4
Cldn12: claudin 12
LEfSe: linear discriminant analysis effect size
PCoA: principal coordinate analysis
CON: control group
POS: positive control group
LOP, model group: received loperamide
LCC: LCC group (received loperamide and low dosage of CCAE)
MCC: MCC group (received loperamide and medium dosage of CCAE)
HCC: HCC (received loperamide and high dosage of CCAE).
