# Supplementary material for: Cymbopogon citratus (DC.) Stapf aqueous extract ameliorates loperamide-induced constipation in mice by promoting gastrointestinal motility and regulating the gut microbiota
Source: Front Microbiol. 2022 Oct 4;13:1017804. doi: 10.3389/fmicb.2022.1017804 (PMC9578511; doi:10.3389/fmicb.2022.1017804)
Supplement: Supplementary file 1 [file Table_1.docx]

**Supplementary Table 1. The main nutritional components of *Cymbopogon citratus* aqueous extract (CCAE)**

| Composition | Content (%) | Method | Reference |
| --- | --- | --- | --- |
| Moisture | 4.06 | Direct drying | [1] |
| Ash | 13.52 | Weigh after burning | [2] |
| Fat | 1.99 | Acid-hydrolysis | [3] |
| Protein | 8.54 | Kjeldahl | [4] |
| Dietary fiber | 11.79 | Enzyme gravimetric | [5] |
| Carbohydrate | 60.10 | Calculated | [6] |
| Sodium | 0.11 | Flame atomic absorption spectroscopy | [7] |
| Total acid | 2.57 | pH potentiometric titration | [8] |
| Polysaccharide | 0.65 | Spectrophotometry | [9] |
| Phytochemical composition | - | HPLC-QQQ-MS/MS | [10] |

**Reference:**

[1] GB 5009.3-2016, National food safety standard-Determination of moisture in food[S]. (In Chinese)

[2] GB 5009.4-2016, National food safety standard-Determination of ash in food[S]. (In Chinese)

[3] GB 5009.6-2016, National food safety standard-Determination of fat in food[S]. (In Chinese)

[4] GB 5009.5-2016, National food safety standard-Ddetermination of protein in food[S]. (In Chinese)

[5] GB 5009.88-2014, National food safety standard-Determination of dietary fiber in food[S]. (In Chinese)

[6] GB/Z 21922-2008, Basic terms of food nutrients[S]. (In Chinese)

[7] GB 5009.91-2017, National food safety standard-Determination of potassium and sodium in food[S]. (In Chinese)

[8] GB 12456-2021, National food safety standard-Determination of total acids in foods[S]. (In Chinese)

[9] NY/T 1676-2008, Determination of crude polysaccharide in edible fungi[S]. (In Chinese)

[10] Doppler, M., Kluger, B., Bueschl, C., Schneider, C., Krska, R., Delcambre, S., Hiller, K., Lemmens, M., & Schuhmacher, R. (2016). Stable Isotope-Assisted Evaluation of Different Extraction Solvents for Untargeted Metabolomics of Plants. International journal of molecular sciences, 17(7), 1017. https://doi.org/10.3390/ijms17071017.
