# Supplementary material for: Cymbopogon citratus (DC.) Stapf aqueous extract ameliorates loperamide-induced constipation in mice by promoting gastrointestinal motility and regulating the gut microbiota
Source: Front Microbiol. 2022 Oct 4;13:1017804. doi: 10.3389/fmicb.2022.1017804 (PMC9578511; doi:10.3389/fmicb.2022.1017804)
Supplement: Supplementary file 2 [file Table_2.docx]

Supplementary Table 2. Primers sequences used for quantitative PCR analysis of gene expression

| Primer | Sequence (5' to 3') |
| --- | --- |
| *RPL-19* | GAAGGTCAAAGGGAATGTGTTCA |
|  | CCTTGTCTGCCTTCAGCTTGT |
| *SCF* | CTCAGTTTTGTGGCTTCGTTTA |
|  | CTACCATGTCCGATACTACGAC |
| *c-Kit* | CAGAAACCCATGTATGAAGT |
|  | CTTTCCAAAACTCAGCCTGT |
| *Ano1* | GGTGTCGGGTTTGTGAAGAT |
|  | TGCACGTTGTTCTCTTCAGG |
| *RyR3* | GGCCAAGAACATCAGAGTGACTAA |
|  | TCACTTCTGCCCTGTCAGTTTC |
| *smMLCK* | AGAAGTCAAGGAGGTAAAGAATGATGT |
|  | CGGGTCGCTTTTCATTGC |
| *IL-1β* | TCCATGAGCTTTGTACAAGGA |
|  | AGCCCATACTTTAGGAAGACA |
| *IL-10* | CATCGATTTCTTCCCTGTGAA |
|  | TCTTGGAGCTTATTAAAGGCATTC |
| *Muc-2* | ACGTGTCATATTTGCACCTCT |
|  | TCAACATTGAGAGTGCCAACT |
| *Cldn4* | GCAGAGCACAGGTCAGATGCA |
|  | AGGGCAGGTCCTGGAGAATGT |
| *Cldn12* | TGTGTGCAGATGTGCTCCTGT |
|  | GCAGGAGGGCTTGAGCTGTAT |
| *Occludin* | ATGTCCGGCCGATGCTCTC |
|  | TTTGGCTGCTCTTGGGTCTGTAT |
| *ZO-1* | TTTTTGACAGGGGGAGTGG |
|  | TGCTGCAGAGGTCAAAGTTCAAG |
| *ZO-2* | CGAAGCAGTCTGGGTCTCTGA |
|  | CCGGCTCCTCTAGCTCATTGT |
| *Aqp4* | GCAGACAAGGTGCAACGTGGTT |
|  | GGCGGAAGGCAAAGCAGTATGG |
| *IL-6* | GTTCTCTGGGAAATCGTGGA |
|  | TGTACTCCAGGTAGCTA |
| *TNFα* | AGACCCTCACACTCAGATCA |
|  | TCTTTGAGATCCATGCCGTTG |
| *MCP* | TTAAAAACCTGGATCGGAACCAA |
|  | GCATTAGCTTCAGATTTACGGGT |
| *Defa* | GGTGATCATCAGACCCCAGCATCAGT |
|  | AAGAGACTAAAACTGAGGAGCAGC |
| *Lyz1* | GCCAAGGTCTACAATCGTTGTGAGTTG |
|  | CAGTCAGCCAGCTTGACACCACG |
