# Supplementary material for: Cymbopogon citratus (DC.) Stapf aqueous extract ameliorates loperamide-induced constipation in mice by promoting gastrointestinal motility and regulating the gut microbiota
Source: Front Microbiol. 2022 Oct 4;13:1017804. doi: 10.3389/fmicb.2022.1017804 (PMC9578511; doi:10.3389/fmicb.2022.1017804)
Supplement: Supplementary file 3 [file Table_3.docx]

**Supplementary Table 3. Classification of Chemical compounds of CCAE**

| Classification of compounds | Relative abundance (%) |
| --- | --- |
| Flavonoids | 24.30 |
| Nucleotide | 19.66 |
| Amino acid | 15.81 |
| Organooxygen compounds, Carboxylic acids | 14.85 |
| Alkaloids | 13.51 |
| Lipids, Benzene and substituted derivatives | 3.89 |
| Phenols | 2.40 |
| Vitamins, Organic acids | 1.18 |
| Carbohydrates, polyol | 0.83 |
| Terpenoids | 0.83 |
