# Supplementary material for: Cymbopogon citratus (DC.) Stapf aqueous extract ameliorates loperamide-induced constipation in mice by promoting gastrointestinal motility and regulating the gut microbiota
Source: Front Microbiol. 2022 Oct 4;13:1017804. doi: 10.3389/fmicb.2022.1017804 (PMC9578511; doi:10.3389/fmicb.2022.1017804)
Supplement: Supplementary file 4 [file Table_4.docx]

**Supplementary Table 4. Chemical compounds of CCAE**

| Compounds | Class | Relative abundance  (%) |
| --- | --- | --- |
| Betaine | Alkaloids | 13.58 |
| Pyrrolidonecarboxylic acid | Carboxylic acids | 9.91 |
| Vidarabine | Purine nucleosides | 7.45 |
| Proline | Amino acid | 6.50 |
| Homoorientin | flavonoids | 5.74 |
| Cyanidin 3-rutinoside;Biorobin | Flavonoids | 4.71 |
| Vitexin | Flavonoids | 3.42 |
| Benzocaine | Benzene and substituted derivatives | 2.08 |
| Guanine | Nucleotide | 1.86 |
| Phenylalanine | Amino acid | 1.80 |
| Crotonoside | Alkaloids | 1.73 |
| Cordycepin | Nucleotide | 1.71 |
| Adenosine 2',3'-cyclic phosphate | Purine nucleotides | 1.61 |
| Guanosine 3',5'-cyclic monophosphate | Nucleotide | 1.53 |
| 5'-Deoxyadenosine | Nucleotide | 1.49 |
| Cynaroside | Flavonoids | 1.45 |
| D-alpha-Aminobutyric acid | Carboxylic acids | 1.35 |
| Rutin | Flavonoids | 1.32 |
| 2-Phenylacetamide | Benzene and substituted derivatives | 1.14 |
