# Supplementary figures and images for: Cymbopogon citratus (DC.) Stapf aqueous extract ameliorates loperamide-induced constipation in mice by promoting gastrointestinal motility and regulating the gut microbiota
Source: Front Microbiol. 2022 Oct 4;13:1017804. doi: 10.3389/fmicb.2022.1017804 (PMC9578511; doi:10.3389/fmicb.2022.1017804)

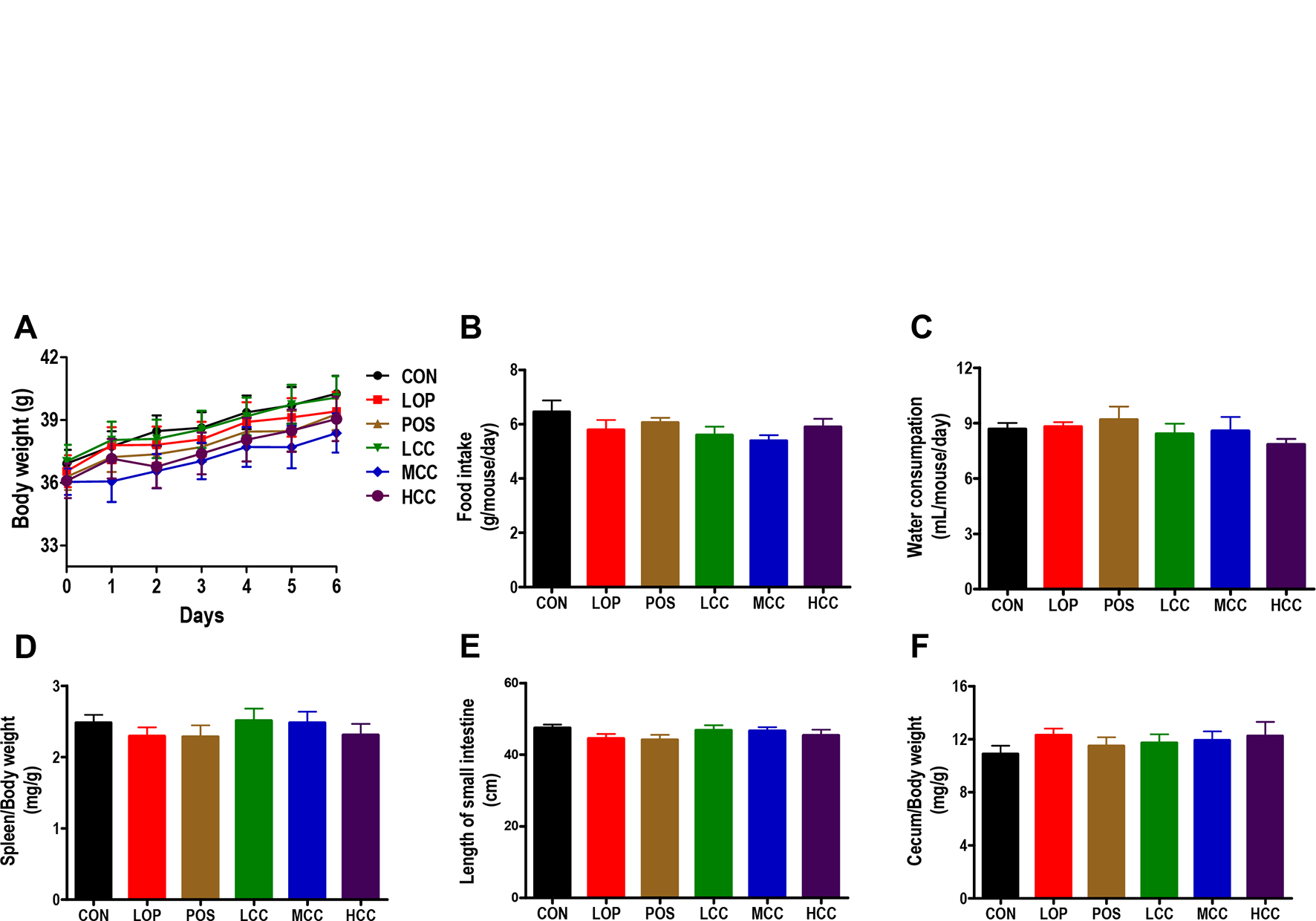

Supplement: Supplementary Figure 1 — Effects of CCAE on loperamide-induced constipation symptoms in mice. (A) The body weight. (B) Food intake. (C) The water consumption. (D) Spleen index. (E) The small intestine length. (F) Cecum index. [file Image_1.TIF]

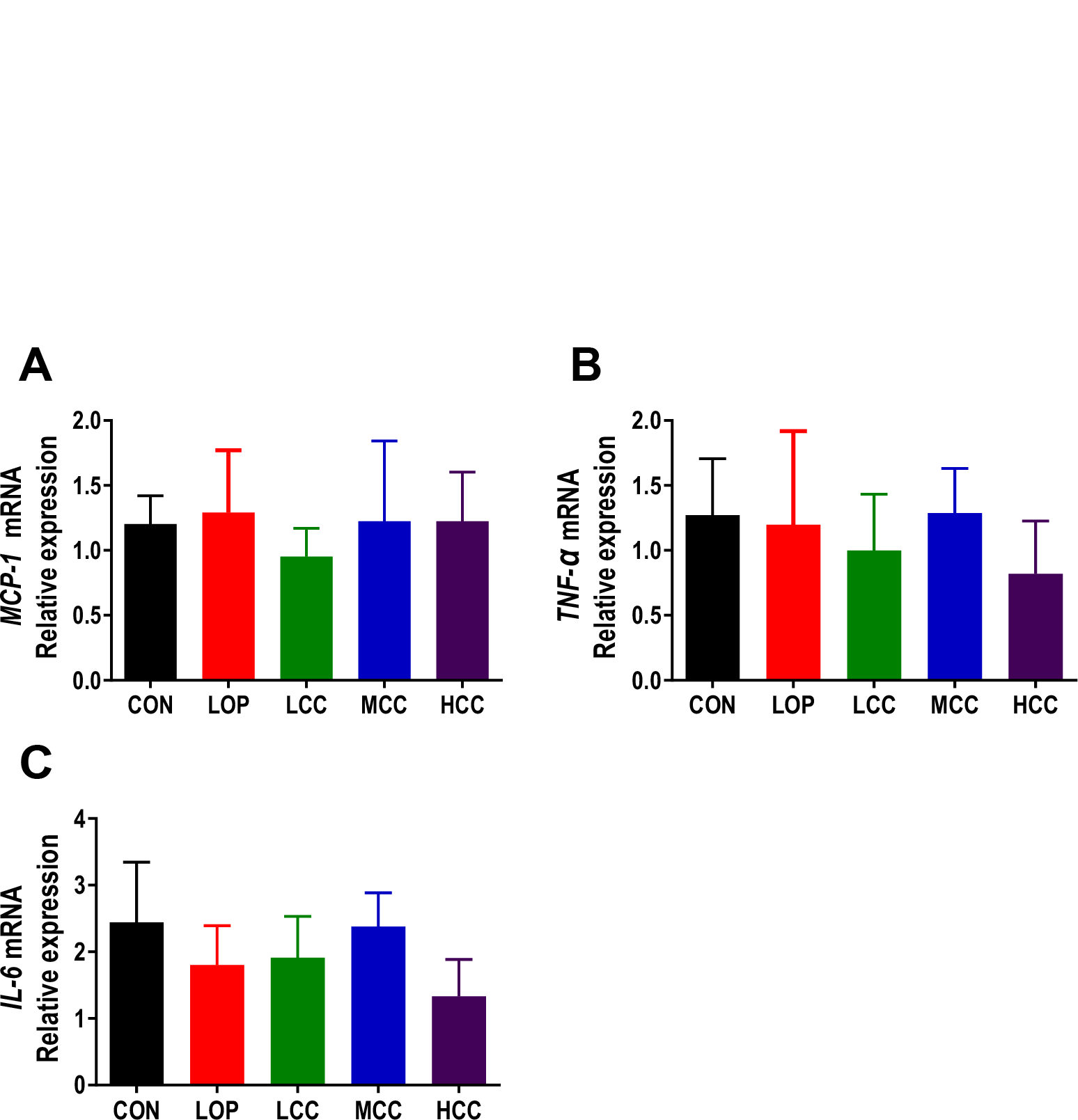

Supplement: Supplementary Figure 2 — Gene expression of intestinal inflammatory factors in colon of STC mice. (A–C) Inflammatory factor MCP-1, TNF-α, and IL-6. [file Image_2.TIF]

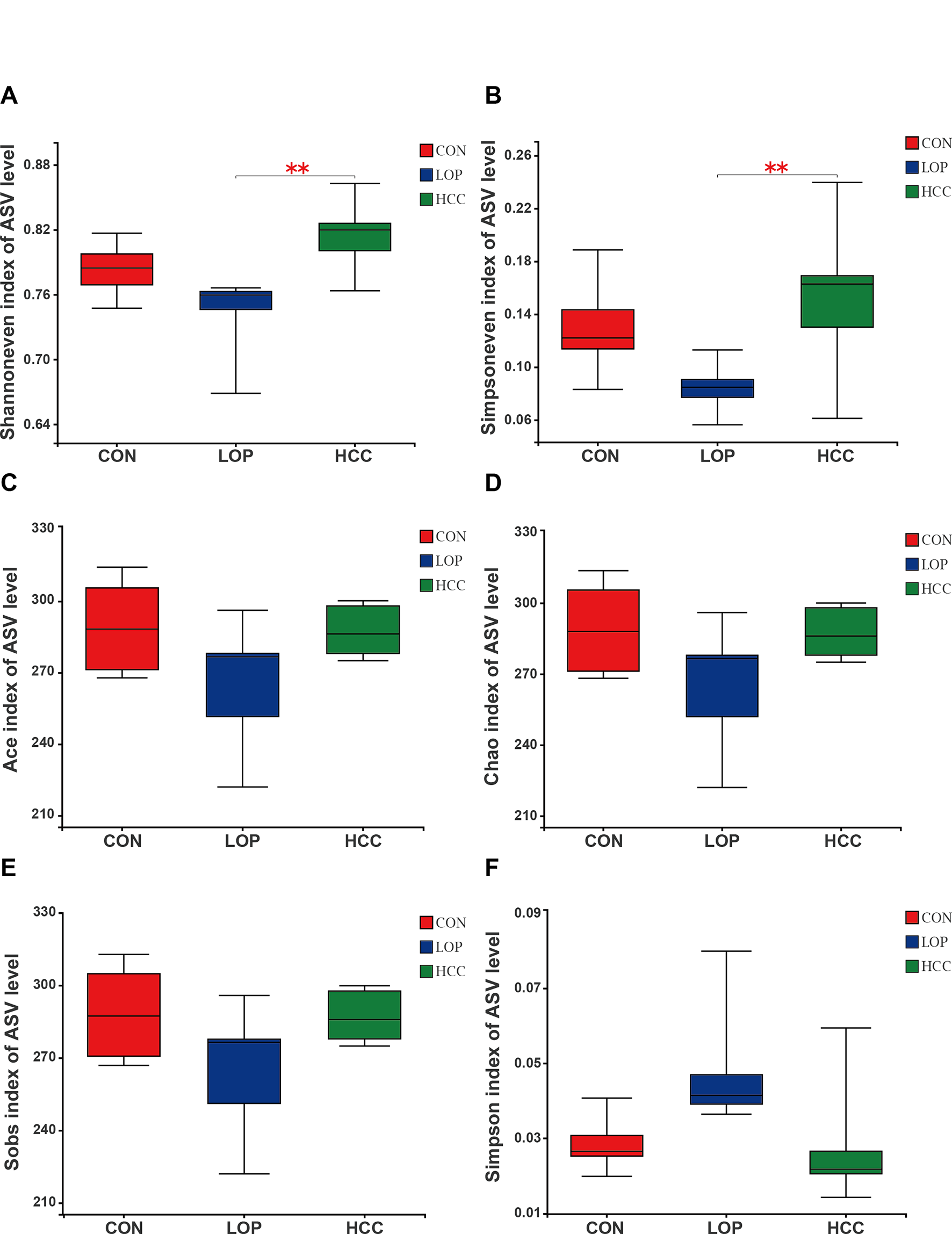

Supplement: Supplementary Figure 3 — (A–F) Shannoneven, simpsoneven, ace, chao, sobs, simpson index of ASV level. *compared with the NCD group, using the Kruskal-Wallis H test. **P < 0.01. [file Image_3.TIF]

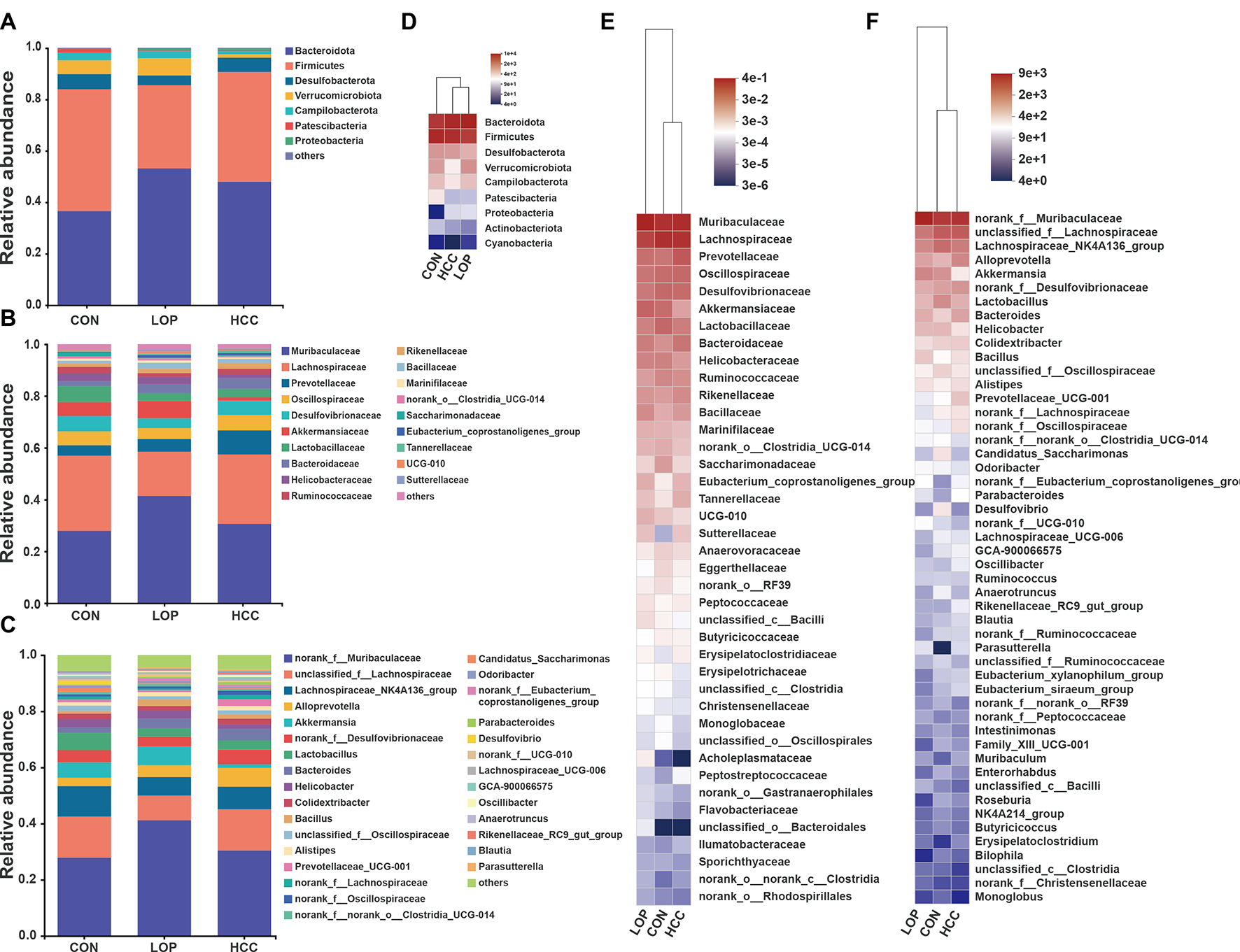

Supplement: Supplementary Figure 4 — Effect of CCAE on the cecum microbial composition in STC mice. (A) Phylum level. (B) Family level. (C) Genus level. (D–F) Cluster heatmaps of gut microbiota in different groups. [file Image_4.TIF]

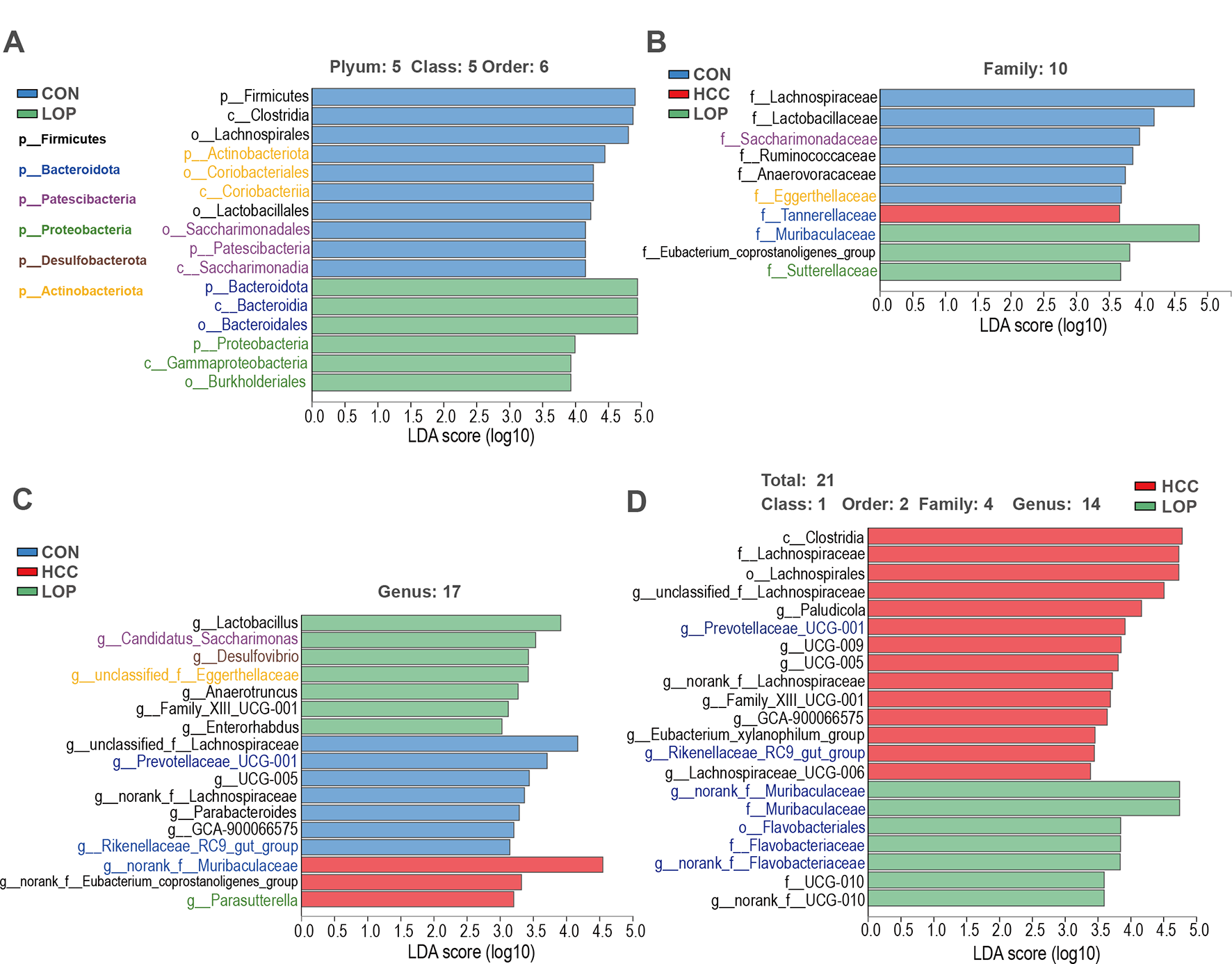

Supplement: Supplementary Figure 5 — Linear discriminant analysis effect size (LEfSe) analyses (LDA score > 2.0). (A–C) LEfSe analyses based on the CON, LOP and HCC groups. (A) At the phylum, class and order level. (B) At the family level. (C) At the genus level. (D) LEfSe analyses based on the LOP and HCC groups, from the phylum level to the genus level. [file Image_5.TIF]

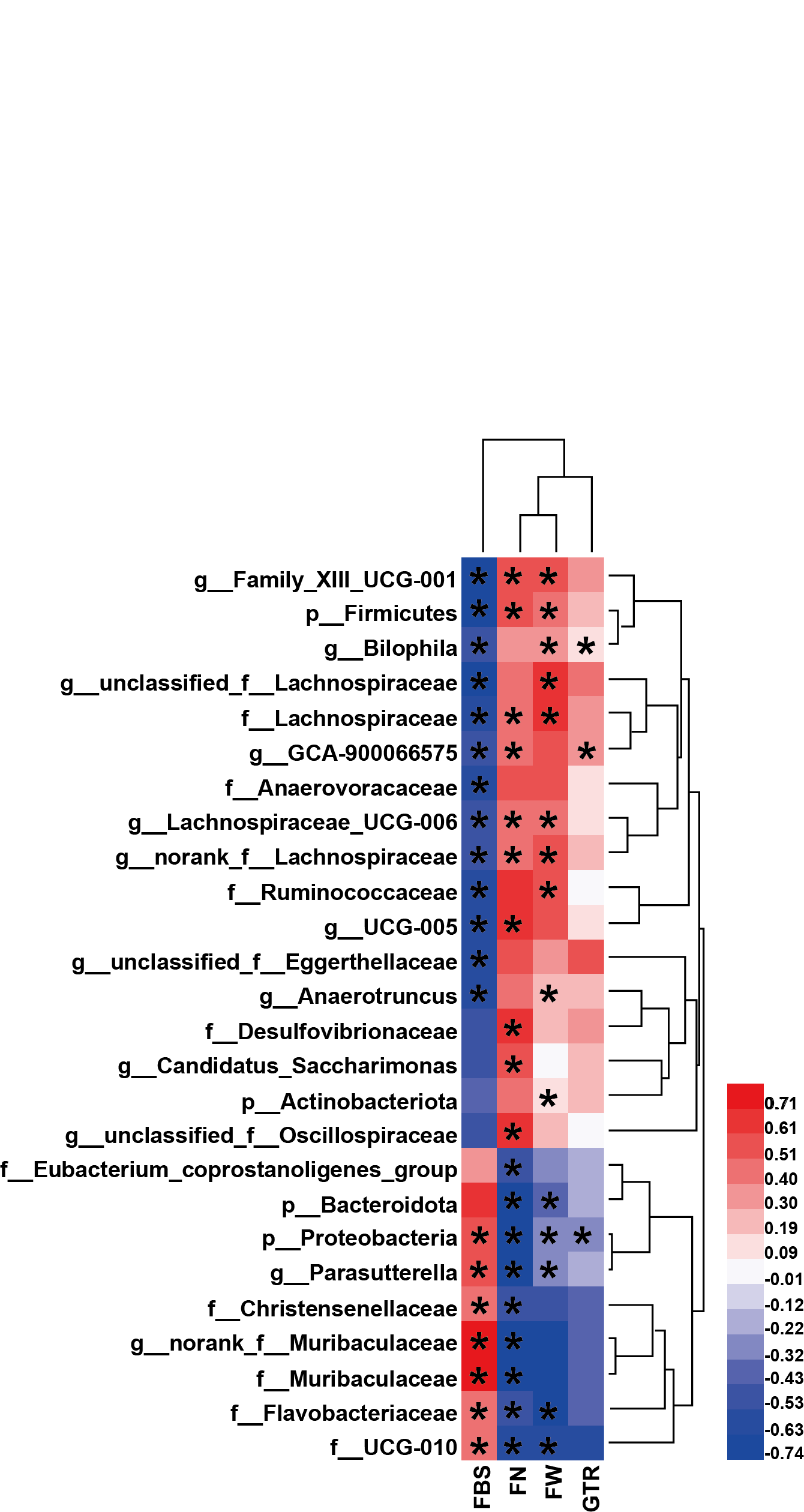

Supplement: Supplementary Figure 6 — Heat maps showing correlations between specific gut bacteria and the core laxative phenotypic indicators in STC mice. Bivariate correlations (P < 0.05, n = 6 in each group), including correlations between gut bacteria and the core laxative phenotypic indicators. FBS, the defecation time of the first black stool; the fecal wet weight (FW) and the fecal number (FN) in 6 h; the gastrointestinal transit rate (GTR). The color at each intersection indicates the value of the r coefficient; P-values were adjusted for multiple testing according to the Bonferroni and Hochberg procedures. * indicates a significant correlation between these two parameters (P < 0.05). [file Image_6.TIF]
